# Real-time in vivo imaging of subpopulations of circulating tumor cells using antibody conjugated quantum dots

**DOI:** 10.1186/s12951-019-0453-7

**Published:** 2019-02-06

**Authors:** Chiung Wen Kuo, Di-Yen Chueh, Peilin Chen

**Affiliations:** Research Center for Applied Sciences, Academia Sinica, Taipei, Taiwan

**Keywords:** Multiphoton imaging, Quantum dots, Circulating tumor cells, Cancer stem cells

## Abstract

**Introduction:**

The detection of circulating tumor cells (CTCs) is very important for cancer diagnosis. CTCs can travel from primary tumors through the circulation to form secondary tumor colonies via bloodstream extravasation. The number of CTCs has been used as an indicator of cancer progress. However, the population of CTCs is very heterogeneous. It is very challenging to identify CTC subpopulations such as cancer stem cells (CSCs) with high metastatic potential, which are very important for cancer diagnostic management.

**Results:**

We report a study of real-time CTC and CSC imaging in the bloodstreams of living animals using multi-photon microscopy and antibody conjugated quantum dots. We have developed a cancer model for noninvasive imaging wherein pancreatic cancer cells expressing fluorescent proteins were subcutaneously injected into the earlobes of mice and then formed solid tumors. When the cancer cells broke away from the solid tumor, CTCs with fluorescent proteins in the bloodstream at different stages of development could be monitored noninvasively in real time. The number of CTCs observed in the blood vessels could be correlated to the tumor size in the first month and reached a maximum value of approximately 100 CTCs/min after 5 weeks of tumor inoculation. To observe CTC subpopulations, conjugated quantum dots were used. It was found that cluster of differentiation (CD)24+ CTCs can move along the blood vessel walls and migrate to peripheral tissues. CD24+ cell accumulation on the solid tumors’ sides was observed, which may provide valuable insight for designing new drugs to target cancer subpopulations with high metastatic potential. We also demonstrated that our system is capable of imaging a minor population of cancer stem cells, CD133+ CTCs, which are found in 0.7% of pancreatic cancer cells and 1%–3% of solid tumors in patients.

**Conclusions:**

With the help of quantum dots, CTCs with higher metastatic potential, such as CD24+ and CD133+ CTCs, have been identified in living animals. Using our approach, it may be possible to investigate detailed metastatic mechanism such as tumor cell extravasation to the blood vessels. In addition, the number of observed CTCs in the blood stream could be correlated with tumor stage in the early stage of cancer.

**Electronic supplementary material:**

The online version of this article (10.1186/s12951-019-0453-7) contains supplementary material, which is available to authorized users.

## Introduction

One of the major complications for cancer patients is metastasis, which accounts for more than 90% of cancer-related mortality [1–4]. However, our understanding of metastasis is far from complete. It is now commonly believed that some tumor cells disseminated from primary tumors could invade the blood vessels, circulate in the bloodstream and reach distant organs via extravasation. After adapting to the new microenvironment, these surviving tumor cells start to proliferate, forming metastatic sites [3, 4]. During this process, the disseminated tumor cells are in circulation and are termed circulating tumor cells (CTCs). The population of CTCs in blood is extremely low; on average, approximately one CTC can be found in one billion peripheral blood cells [5, 6]. Early studies had indicated that the number of CTCs can be used as a prognostic biomarker for various cancers [7, 8]. However, recent results revealed that the population of CTCs is very heterogeneous; while some CTCs exhibited epithelial phenotypes, other CTCs undergo an epithelial-to-mesenchymal transition (EMT) [6]. In addition, CTCs were found to be capable of clustering with other blood cells such as platelets and leukocytes. It was found that the observation of CTC clusters in the blood could increase metastatic potential in both animals and patients [9, 10]. One major challenge in CTC research concerns the origin of CTCs, which is still debatable. It is very difficult to determine whether the CTCs isolated from peripheral blood are from the primary tumor trying to form metastatic sites or from the established metastatic sites. It is also difficult to determine whether cells shred form tumors without metastatic potential or are just tumor cells responding to the therapy [5]. In other words, there are many CTC subpopulations with genomic, proteomic, and functional differences. Therefore, to use CTCs as a prognostic biomarker, it is very important to study the spatiotemporal behavior of CTCs and identify a specific CTC subpopulation in the bloodstream.

In order to detect or isolate CTCs, the recognition of CTCs by surface markers in which epithelial markers (such as epithelial cell adhesion molecules [EpCAM]) is often necessary. Currently, there is only one Food and Drug Administration (FDA)-approved CTC detection platform, the CellSearch system, which utilizes EpCAM antibody-modified magnetic beads to separate CTCs from other blood cells [11]. Immunofluorescence signals are used to confirm CTCs, which are cytokeratin (CK)-positive and cluster of differentiation (CD)45-negative. It has been shown that patients with more than five CTCs in 7.5 ml of blood had shorter progression-free survival rates [7]. Since the number of CTCs can be correlated to patient status, many recent research efforts have focused on developing CTC separation techniques to enhance isolation efficiency [11]. These techniques rely on the differences in CTCs’ physical properties or surface markers in which microfluidic devices [12–14], filters [15], nanostructures [16–18], and electric or magnetic fields [19, 20] have been used to separate CTCs. In order to increase specificity, surface markers other than EpCAM, including epidermal growth factor receptor (EGFR), human epidermal growth factor (HER)2, and *N*-cadherin, have been used [6]. The results from these studies are very promising and suggest that CTC enumeration can be used to evaluate therapeutic efficacy or even for early cancer detection. Currently, the enumeration of CTCs is often used as a liquid biopsy for cancer management [11]. Isolated CTCs can also be used for genetic analysis for precision medicine. However, the specificity of these isolation techniques relies on the surface markers. If CTCs do not exhibit these markers, the results may be misleading.

Because tumor cells are very heterogeneous, analyzing CTCs at a single time point may not reveal the whole story. In order to better understand metastases, in vivo imaging has been used to continuously monitor primary tumors and CTCs [21–24]. These in vivo studies allow us to understand the origin and properties of CTCs and the metastatic process. In an early attempt to measure metastasis, green fluorescent protein (GFP)-labeled tumor cells were studied using time-lapse confocal imaging, and it was found that tumor cells polarized toward blood vessels [21]. However, the images were taken each minute, which prevented the study of dynamic processes. In order to increase the time resolution, in vivo flow cytometry (IVFC) was used to enumerate CTCs at points in which the number of CTCs in the blood vessels could be counted [22]. In order to further quantify CTCs, multiphoton intravital flow cytometry, which is capable of quantifying 2 CTCs/ml in blood, was developed [23]. In order to increase the speed, line scans were used to enumerate CTCs, which leads to the loss of CTC spatial information. In addition, CTCs were labeled with folate conjugated dyes, which cannot be used for CTCs without folate receptors. In order to observe the formation of metastasis, fluorescent-labeled tumor cells were injected into animal tail veins or arteries [24–27]. The behavior of CTCs in the bloodstream can be investigated using real-time imaging. However, cancer cells observed in the circulation using such an approach had not originated from primary tumors. The behavior of the injected cancer cells in circulation may be different from spontaneous metastasis models. For example, the number of CTCs measured in such a model had no correlation with solid tumors, and the CTC population was rather homogenous.

In this study, we present the study of CTCs’ real-time behavior using multiphoton microscopy. We have established a spontaneous circulating tumor cell model using cancer cells expressing fluorescent proteins on the earlobes of mice in which the circulating tumor cells exhibiting fluorescence signals can be monitored in the bloodstream noninvasively at various stages of tumor growth. The numbers, velocities, and trajectories of CTCs in the bloodstream can be measured concurrently. The microenvironment around the tumor mass can also be investigated via second harmonic generation. Since CTCs exhibit unique fluorescence signals, we can study a CTC subpopulation using antibody conjugated quantum dots with emissions at different wavelengths. This information is very important for gaining an understanding of tumor cell metastasis.

## Results

### Validation of tumor model on the mouse earlobes

For long-term in vivo imaging, window chambers are normally installed in the animals [28]. However, such invasive procedures may lead to complications in animals. Therefore, we adapted a model developed by Matsumoto et al. [29] wherein tumors are grown on the earlobes of mice to allow the direct imaging of tumors via multiphoton microscopy. In order to evaluate the capability of our system for real-time noninvasive blood vessel imaging, we first imaged the blood vessels in the earlobes of mice using Evans blue, which was administered via tail vein injection. A very low fluorescence background was observed in the blood vessels before the arrival of the Evans blue dye. A clear increase in the fluorescence intensity could be seen in a 50 µm blood vessel 12 s after injection, and an additional 20 s were needed for the Evans blue to reach nearby blood vessels with a diameter of 100 µm (Additional file 1: Figure S1). The results shown in the figure clearly indicate that our system can noninvasively image blood vessels in living animals.

In order to create tumors on the earlobes of mice, 20 μl of fluorescent-protein-labeled cancer cells at a concentration of 10^6^ cell/ml were injected into the earlobes of a mouse. Solid tumors on the inoculation site were seen 2 weeks after injection, and the volume of the solid tumors increased with time. When red fluorescent-protein (RFP)-labeled cancer cells were used in the experiment, the solid tumors on the earlobes of the mice were clearly seen using the Intravital Imaging System (IVIS). Additional file 1: Figure S2a and b show tumors grown on the earlobes of mice 8 weeks after subcutaneous injection. Metastatic sites were found in the stomach and intestines as shown in Additional file 1: Figure S2c, indicating that our model underwent a metastasis process. Recently, it has been shown that extravasation often takes place in small capillaries, which was found to be an important finding concerning tumor cell transmigration [3]. The average diameter of small capillaries in our tumor model was measured to be approximately 5 µm (Additional file 1: Figure S3), which was comparable to the reported value [4]. In addition to blood vessels in solid tumors, collagen fibers are an important feature of the tumor microenvironment. We analyzed the width of the cross-linked collagen fibers in the extracellular matrix surrounding tumors using the second harmonic generation. Fibrillar collagen networks can be classified into two categories: (1) elongated or (2) curled (Additional file 1: Figure S4). The average width of curled fibers was measured to be < 2 µm, whereas that of the elongated fibers was approximately 4 µm.

### In vivo imaging for monitoring circulating tumor cells

**Fig. 1:**
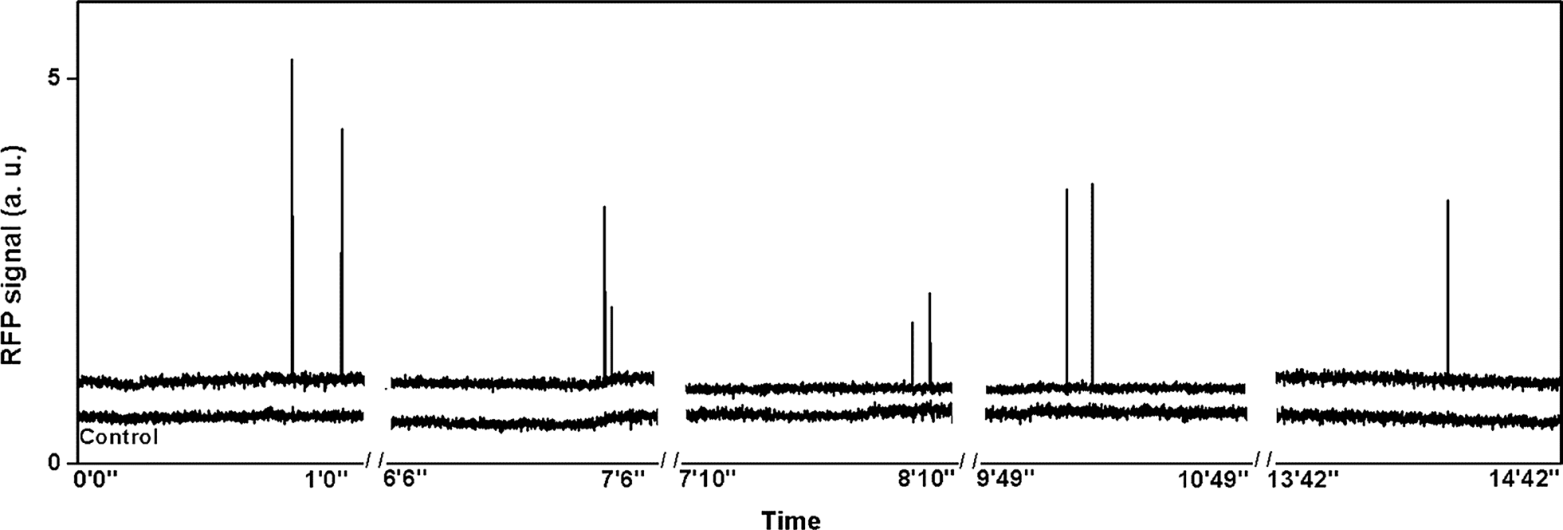
Integrated fluorescence signals from a section of a blood vessel with a diameter of 50 µm as a function of observation time. The spikes in integrated fluorescence intensity indicate that individual circulating tumor cells (CTCs) are passing through the detection region. Bottom control signals are the integrated fluorescence signals from a section in the same blood vessel without detectable CTCs

**Fig. 2:**
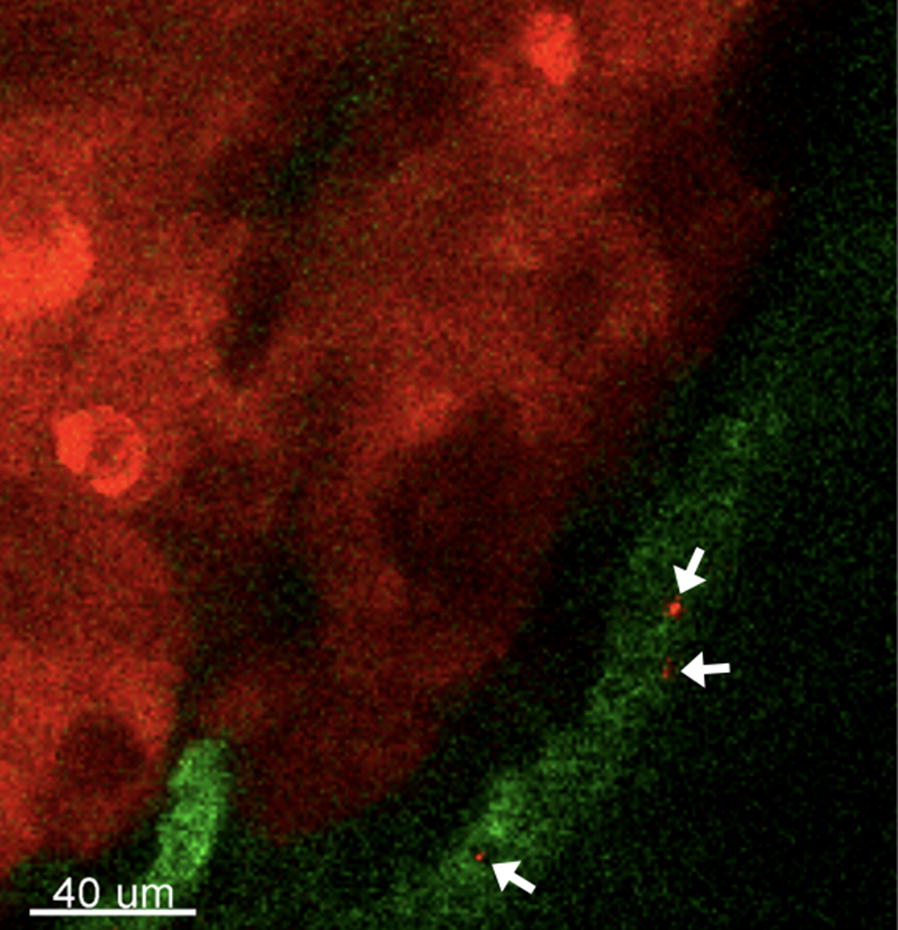
CTCs (red cells indicated by arrows) in the blood vessel near solid tumor expressing red fluorescent protein (RFP). The blood vessels (green) were stained with fluorescein isothiocyanate (FITC)-dextran. Tumor cells: BXPC3-RFP

**Fig. 3:**
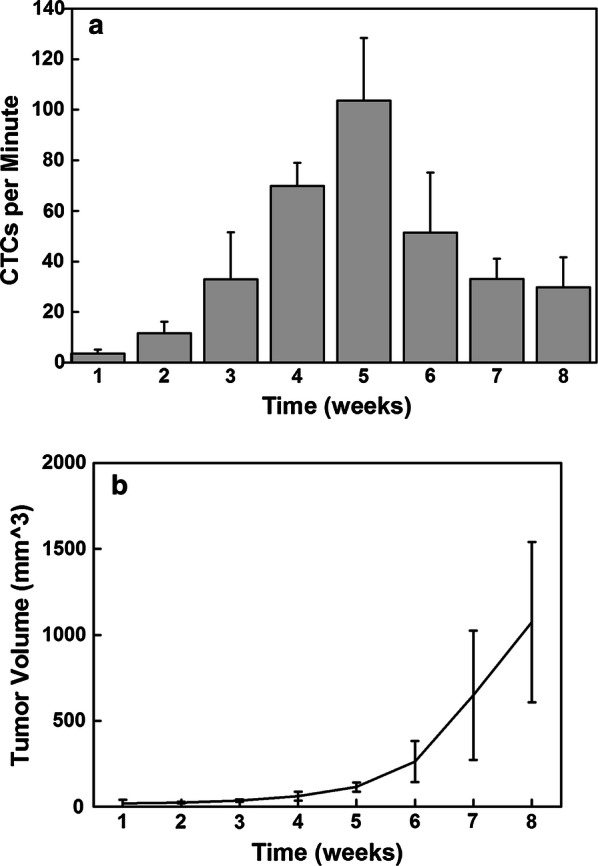
**a** The number of detected CTC/min in the blood vessels near the solid tumors on the earlobe at different time points post-inoculation. **b** The volume of solid tumors at different time points (n = 3)

**Fig. 4:**
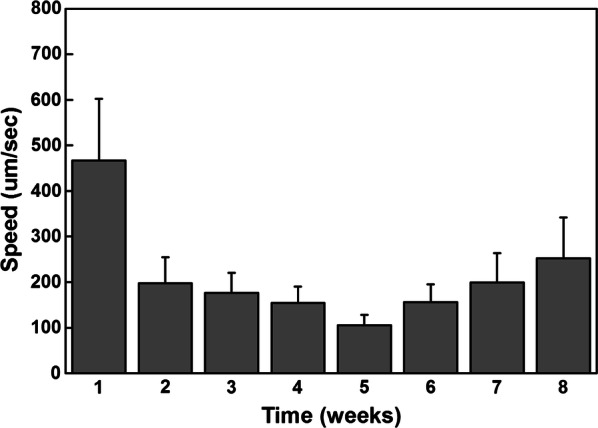
The average velocity of observed CTCs after tumor inoculation over time

For the observation of circulating tumor cells, BXPC3 cells expressing RFP were used. After 1 week of inoculation, CTCs could be observed in the blood vessels near the solid tumor. In order to mimic the in vivo flow cytometry, we integrated fluorescence intensity over a blood vessel’s cross-section from the real-time images as shown in Fig. 1 in which the control data were obtained from a region at the edge of the same blood vessels without detectable CTCs. Since the CTC population was low, spikes in the integrated fluorescence intensity can be seen occasionally, indicating the passage of individual CTCs through the detection region. Using the real-time images, we could monitor the trajectories of individual CTCs, which allowed us to count the number of CTCs in the blood vessels. As the tumor grew, the number of observed CTCs increased. The CTCs moving in a blood vessel near solid tumor where tumor cells expressing RFP allowed the visualization of solid tumor and CTCs are shown in Fig. 2. As shown in Fig. 3, the detected number of CTCs per minute could be correlated with the size of the solid tumors (Fig. 3b) in the first 5 weeks (results for individual mice can be found in Additional file 1: Figure S5). The observed number of CTCs in blood vessels reached a maximum value of approximately 100 CTC/min. Although the size of tumors continued to increase, the number of CTCs decreased after 6 weeks. At week 8, the number of detected CTCs in the blood vessels near solid tumors decreased to 20 to 29 CTC/min. The animals were sacrificed after 8 weeks. Using the time-lapse images of CTCs in circulation, we could calculate the velocity of individual CTCs. The average CTC velocity at different stages is shown in Fig. 4. The average CTC velocity at week 1 was the highest (approximately 0.5 mm/s). However, the average velocity decreased to 0.2 mm/s after week 2 and remained roughly the same velocity within experimental error until week 8.

### Imaging CD24+ CTC subpopulation

**Fig. 5:**
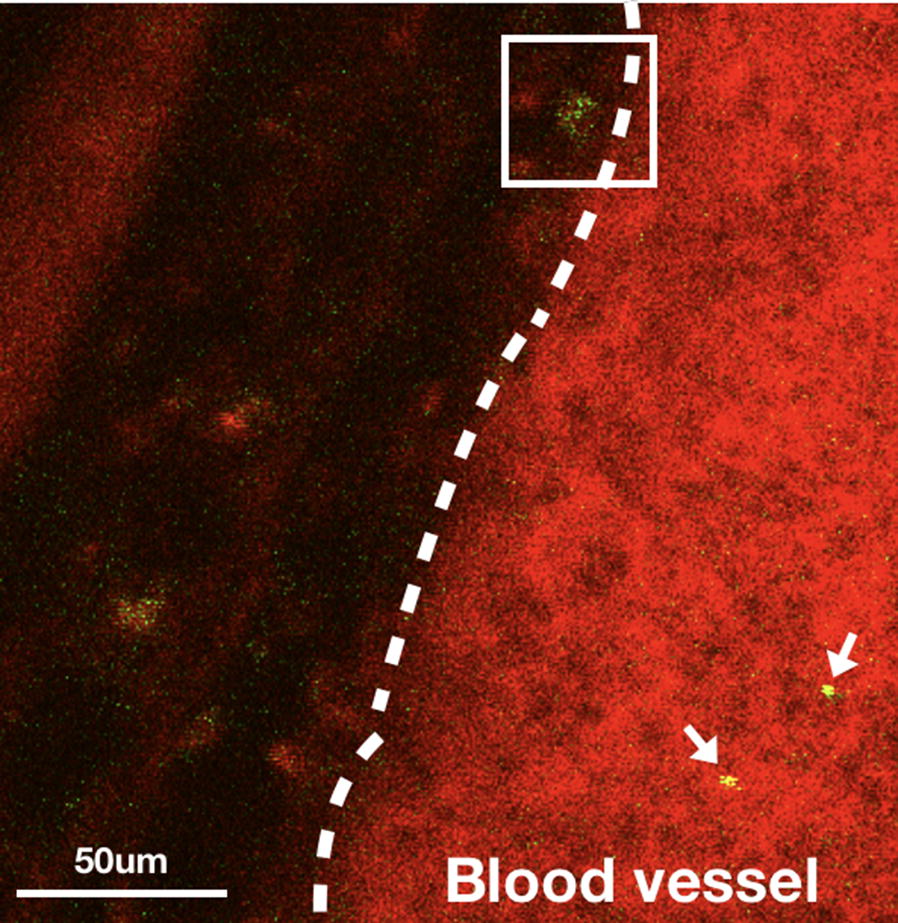
Two cluster of differentiation (CD)24+ CTCs (green, indicated by arrows) are moving in a blood vessel (red) while one CD24+ CTC is moving slowly along the sidewall (highlighted area). The movie can be found in Additional file 3: Movie M2a and Additional file 4: Movie M2b. Accumulation of CD24+ CTCs can be found outside of a blood vessel. Tumor cells: BXPC3-RFP

In order to image a subpopulation of CTCs, we first used monoclonal CD24 antibodies conjugated on the quantum dots (Qdot 525, Invitrogen) to label the CSCs in the bloodstream. Anti-CD24 conjugated quantum dots (100 μl, 0.2 μM) were administered via tail vein injection. This allowed us to monitor a subpopulation of CTCs with the CD24 phenotype. At this concentration, no toxicity of quantum dots was found in mice for several weeks [30]. From Fig. 5, it can be seen that individual CD24+ CTCs (green) can be visualized in the blood vessels, which were labeled with Evans Blue (red). A movie of CD24+ CTCs in the blood vessels can be found in Additional file 2: Movie M1. In this experiment, we found that CD24+ cells travelled rapidly in peripheral vasculatures as shown in Additional file 3: Movie M2a. Occasionally, we found that CTCs crawled along the outer wall of blood vessels and transmigrated to the cancer tissues in proximity as shown in the highlighted area in Fig. 5 and Additional file 4: Movie M2b.

**Fig. 6:**
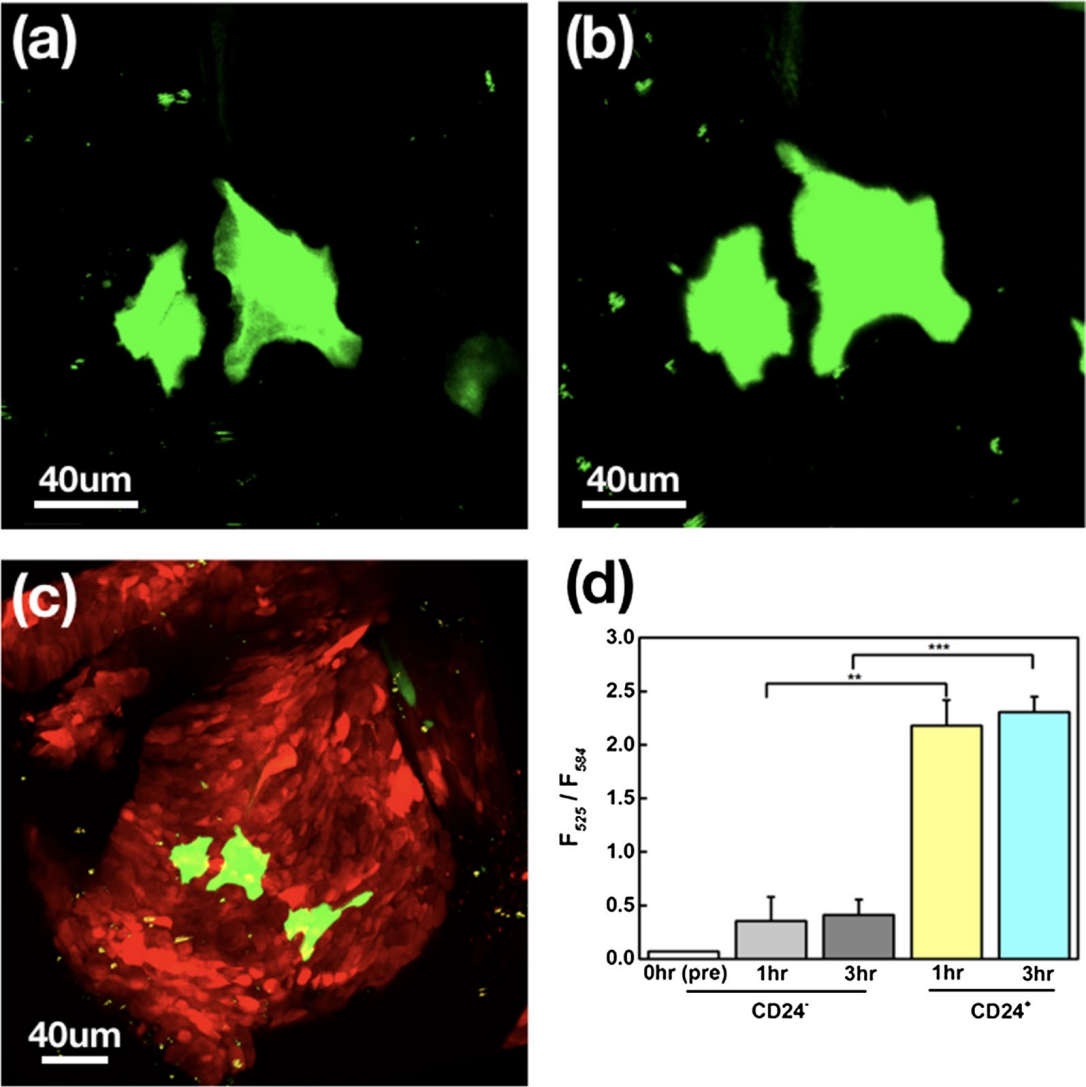
Accumulation of CD24+ signals on the solid tumor **a** 1 h, **b** 3 h after injecting the anti-body conjugated quantum dots. **c** Location of CD24+ cells (green) on the solid tumor (red). **d** The normalized intensity of quantum dots on the solid tumor with and without anti CD24 conjugation at different time points. CD24− data were collected using quantum dots without anti CD24 antibodies. Tumor cells: BXPC3-RFP

The CD24+ cancer cells were found not only in the peripheral tumor tissues but also on the solid tumor. Accumulation of CD24+ cells can be found in solid tumors 1 h after injection of CD24 antibody-coated quantum dots. The accumulation of green fluorescence signals was found on the solid tumor (red) as shown in Fig. 6. In order to quantify the accumulation of CD24+ cells on the solid tumor, we compared the fluorescence intensity ratio (F525/F584) changes at 1 and 3 h after quantum dot injection. The control experiment was carried out by injecting the same amount of streptavidin-conjugated quantum dots without CD24 antibody. The results are shown in Fig. 6d. The accumulation of CD24+ cancer cells on only one part of the solid tumor suggests that it is possible to design new drugs to target a subpopulation of cancer cells, for example as in this case, cancer stem cells with higher metastatic potentials.

### Imaging CD133+ CTC subpopulation

**Fig. 7:**
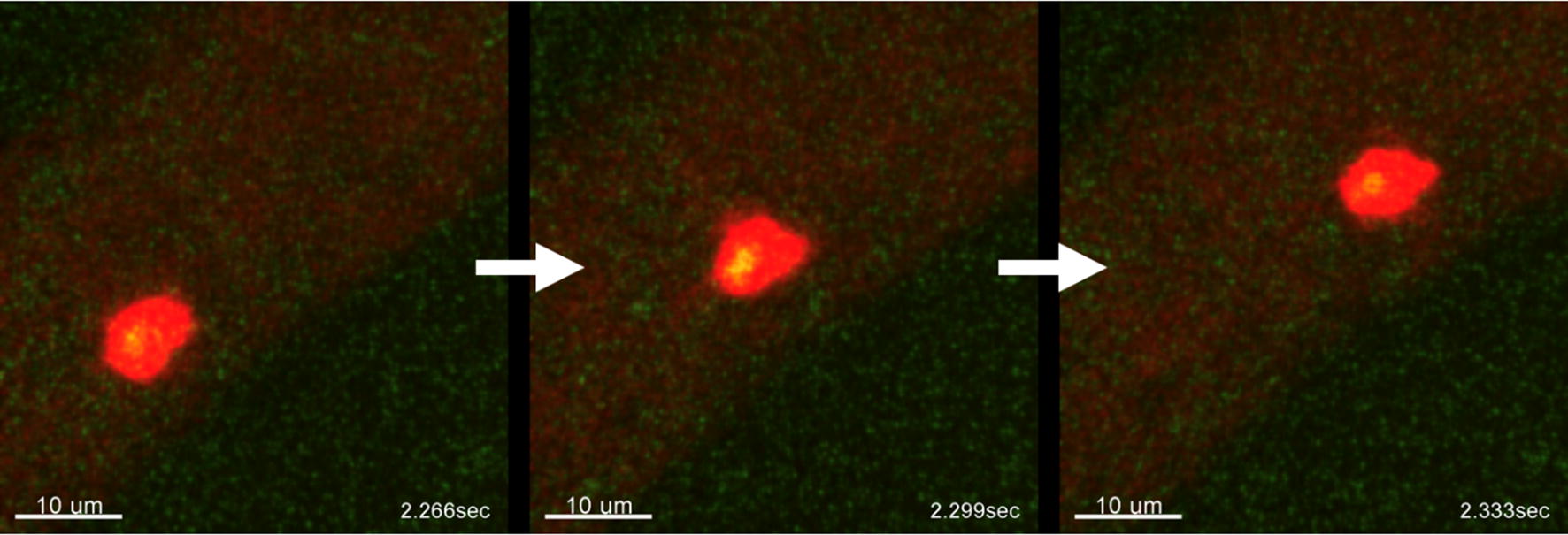
Three consecutive images of CD133+ CTC moving in the blood vessels (time interval: 33 ms). Red signals are from quantum dots and green signals are from tumor cell expressing green fluorescent protein (GFP). The movie can be found in Additional file 5: Movie M3. Tumor cells: BXPC3-GFP

It has been shown that CD133+ cells are rare and exhibit higher metastatic potential [31]. Therefore, we wanted to examine whether our system was sufficiently sensitive for detecting a rare subpopulation of cancer stem cells in the circulation. Indeed, when the quantum dots coated with anti-CD133 were injected into the animal, we were able to observe CTCs with CD133 surface markers. Figure 7 depicts three consecutive images (33 ms interval) of a tumor cell circulating in the bloodstream with fluorescent signals (cancer cells) from green fluorescent proteins (GFP) and anti-CD133 conjugated quantum dots (Qdot 705, Invitrogen). The movement of the circulating tumor cells with CD133 surface markers can be found in the Supplementary Information (Additional file 5: Movie M3). Since the CD133+ CTCs exhibited two colors (green and red), the disappearance and reappearance of the green color on the CTC revealed that CD133+ CTCs were rolling rapidly on the blood vessel. During the observation period, we detected 73 CTCs, only two of which had red florescence signals. This indicates that our approach allowed us to image a very small CTC subpopulation with CSC markers in circulation.

## Discussion

The use of a multiphoton imaging system allowed us to investigate the solid tumor. Detailed structures such as individual tumor cells, blood vessels, and fibers formed by extracellular matrix elements, can also be studied. With the real-time imaging system, we were able to monitor behavior of individual CTCs and their subpopulations. One of the most important issues for the study of CTCs is their enumeration. In general, the number of CTCs in the bloodstream is very low and the lifetime of CTCs is estimated to be 1–2 h [1], which reduces the chance of CTC detection via blood sampling at arbitrary locations. In our system, we monitored CTCs in blood vessels a few hundred micrometers away from solid tumors (the imaging area was located just outside the boundary solid tumor), which were the major source of the circulating CTCs. Fluorescent proteins allowed us to detect CTCs in real-time in the blood vessels. Since some CTCs traveled very rapidly in the blood vessels and may not always remain in the focal planes, we developed criteria for CTC identification: (1) the fluorescence intensity of CTCs should be at least five times higher than the background and (2) CTCs should appear at least in three frames when traveling through the detection region.

In this study, the maximum number of CTCs that we monitored was approximately 100 CTCs/min in a blood vessel near a solid tumor. The detected CTC velocity was approximately 0.1–0.5 mm/s. If we had tried to detect CTCs at random sites far from the solid tumor, the CTC density would have been diluted by several orders of magnitude, which makes real-time sensing almost impossible. Indeed, a recent experiment demonstrated that only approximately 100 CTC/min can be observed at random sites 1 min after injecting 10^6^ cancer cells through the tail vein [32]; this number decreases significantly a few minutes later. From the real-time images, we could capture the image of individual CTCs at video rate (30 Hz), which allowed us to trace the trajectories of CTCs and monitor the location of CTCs in the blood vessels revealing the extravasation process. From the CTC trajectories, we could calculate velocity, which depended on both the CTC properties and the blood vessel geometry. Thus, we should compare the velocity of CTCs at blood vessels of similar diameters and geometries.

The decrease in the CTC number after 6 weeks may be due to the loss of the fluorescent proteins’ fluorescence intensity in some tumor cells. It has been shown that the fluorescence intensity of these proteins decreased significantly under hypoxic conditions [33]. At week 6, the tumor size increased substantially. The oxygen level in the tumor was low, which would have reduced the expression of fluorescent proteins resulting in undetectable CTCs. Therefore, the number of detectable CTCs decreased after week 6; thus, the number of CTCs could be correlated with the tumor size only in the first 5 weeks.

In a previous study, it was shown that the association of the circulating tumor cells with platelets could contribute to a spontaneous metastasis process [34]. The metastatic capability of platelet-associated CTCs has been analyzed quantitatively during transient interactions. In this experiment, we tried to concurrently image CTCs and platelets by labeling the platelets with quantum dots (Qdot 525, Invitrogen) conjugated with anti-CD41 via tail vein injection. We found that the velocities of CTCs and platelets were in the range of 0.2 mm/s in the blood vessels with a width of 50 μm near the tumor (Additional file 1: Figure S6, Additional file 6: Movie M4). We determined that platelets and CTC cells have similar velocities in the bloodstream, which appears to be the same as the blood flow. However, no CTC trajectories were found to overlap with platelet trajectories. Since the CTC population was very low and the labeling efficiency of platelets was not high, it was difficult to observe the association of platelets with CTCs in our experiment’s the time frame. In a previous study, it was found that the percentage of CTC clusters was only about 2.5% of the total CTC population [9].

It is known that the CTC population is very heterogeneous. Some CTC subpopulations play a more important role in metastatic processes. For example, CTC subpopulations with stem cell properties could enter state similar to CSCs. Surface marker, such as CD133, 44, and 24 have been identified and used to enrich of the CSCs in pancreatic cancers [6, 34]. If the behavior of CSCs could be monitored in the bloodstream, it may help us understand the mechanism of metastasis, including the journey through the bloodstream, potential uptake by phagocytic clearance, and extravasation to secondary sites. We first investigated the metastatic process for the CD24+ CTC subpopulation. Labeling the CTCs with antibody-conjugated quantum dots allowed us to perform long-term observation due to their high and robust fluorescence intensity. In Additional file 3: Movie M2a, Additional file 4: Movie M2b and Fig. 5, a CD24+ CTC was found at the edge of a blood vessel and was moving slowly toward the peripheral tissue (highlighted area) while several high-speed CD24+ CTCs passed through the same region. Since the frequency of such extravasation processes is low, it took a long time to directly observe such an event. However, if we waited for a long enough time, several CD24+ CTCs would accumulate on the peripheral tissue. The anti-CD24 antibody-conjugated quantum dots were found not only on individual CTCs but also on the solid tumors as shown in Fig. 6. Since the antibody-conjugated quantum dots were administered via tail vein injection, they could reach the solid tumor though the blood vessels. From the three-dimensional images of the solid tumor (Additional file 7: Movie M5), it can be seen that several blood vessels grew into the solid tumor. The CD24+ subpopulation on the solid tumor might have been the source of CD24+ CTCs observed in the bloodstream. Since CD24+ cells have greater potential for metastasis, our results may provide insight for designing new drugs targeting the CD24+ subpopulation.

Compared with the CD24+ subpopulation, the CD133+ subpopulation is a very small portion of the total number of pancreatic cancer cells. It was found that only 0.7% of BXPC3 cells possess the CD133 surface marker, while 1%–3% of solid tumors from patients were CD133-positive [35]. In this experiment, we observed two CD133+ CTCs out of 73 CTCs. Although the percentage was low, we could still observe them in our system. The percentage was roughly the same as seen in the clinical observation. The detection of a subpopulation of CTCs would allow us to design new experiments to test different hypotheses for the behavior of CSCs in the blood.

## Conclusion

We developed a spontaneous circulating tumor model on the earlobes of mice that allows noninvasive monitoring of CTCs and circulating CSCs in blood vessels near tumors using a multiphoton imaging system. It was found that the number of CTCs in the blood vessels increased as tumor size increased in the first 5 weeks. However, the number of detected CTCs decreased after 5 weeks due to loss of fluorescence intensity of fluorescent proteins under hypoxic conditions. Blood vessels around tumors and their microenvironments have been studied by both multiphoton and second harmonic generation imaging. With the help of antibody-conjugated quantum dots, it is possible to observe CTC subpopulations in blood vessels. We found that CD24+ CTCs could penetrate nearby blood vessels and accumulate in peripheral tissues. The accumulation of CD24+ cells in solid tumors may provide insight for designing new drugs in order to treat tumor subpopulations. Our system also allowed us to monitor a minor subpopulation of CD133+ CTCs. Our approach appears to offer several advantages over other approaches for the study of circulating tumor cells: (1) it is possible to investigate detailed metastasis mechanism steps such as the extravasation of tumor cells to the blood vessels; (2) tumor stage may be accessed by monitoring the number of circulating tumor cells in the early stage of cancer; and (3) rare subpopulations of CTCs with higher metastatic potential such as CSCs can be visualized directly. Even though real-time intravital imaging allows us to image the rare subpopulation of CTCs in circulation, there are still some limitations with respect to this technique. For example, the in vivo labeling efficiency needs to be improved in order to observe rare cell–cell interactions such as a CTC–platelet interaction. The low frequency of rare cell events prevents us from observing metastasis at distant sites, which would take a long time and a lot of data storage space. Because of the optical penetration depth, the applications of this technique to human patients will be limited to tumors (such as skin cancers) that can be reached by laser light.

## Methods

### Cell line

The human pancreatic cancer cell line, BxPC3, expressing RFP or GFP, was used in this experiment. Cells were cultured in Roswell Park Memorial medium (RPMI) 1640 medium supplemented with 10% fetal bovine serum (Invitrogen) and PenStrep (Sigma) and incubated in 5% CO_2_ at 37 °C. Culture media were changed every 3 days. During each passage, BxPC3 cells were washed with phosphate-buffered saline (PBS) twice, and then enzymatically dissociated with 0.05% trypsin ethylenediaminetetraacetic acid (Invitrogen).

### Labeling

In order to visualize the blood vessels, either 0.5% of Evans blue (Sigma-Aldrich) in PBS solution or 40-kDa of fluorescein isothiocyanate (FITC)–dextran solution (Sigma-Aldrich) was injected through the tail vein. In order to label specific cells in circulation, streptavidin conjugated quantum dots (Qdot525, Qdot705) were purchased from Invitrogen. These quantum dots were further conjugated with biotinylated antibodies. The CD41 antibody was obtained from Abcam for the purpose of labeling platelets. In order to label subpopulations of CTCs, CD24 and 133, antibodies were purchased from Miltenyi Biotech (Auburn, CA, USA).

### Animals and spontaneous circulating tumor cell model

Five-week-old male BALC/c nude mice (BioLasco, Taiwan) were allowed to acclimatize for at least 2 weeks before experiments were done. Animals were maintained under specific pathogen-free conditions using standard laboratory procedures. All protocols were approved by the Academia Sinica IACUC. For the spontaneous tumor cell model, 20 μl of BxPC3-RFP or -GFP cells mixed with Matrigel matrix at 1:1 ratio (Corning) were injected into the earlobes of mice at a density of 10^6^/ml. In order to confirm the tumor model and metastasis, the IVIS system (Xenogen) was used to image the whole body of mice 8 weeks after tumor cell injection in a chamber containing a mixture of isoflurane and oxygen. After imaging, the animal was sacrificed. The organs were removed, placed in a Petri dish, and imaged using the same IVIS system (Additional file 1: Figure S2). One key challenge for in vivo CTC study was labelling CTCs with bright fluorophores. In this experiment, flow cytometry was used to sort cancer cells with high fluorescence intensity. Tumor size was measured every week using a digital caliper, and volume was calculated using a modified ellipsoidal formula in which tumor volume = (minor circumference × major circumference)/2. A group of three mice was used for each CTC measurement for 8 weeks.

### Real-time intravital imaging

All real-time images were taken using an Olympus FVMPE RS multiphoton laser canning microscope, which is capable of imaging 512 × 512 pixels at 30 frames per sec. The field of view was 177 μm × 177 μm with a pixel size of 0.34 μm. A MaiTai HP DS-OL laser was used for multiphoton excitation with a tunable wavelength ranging from 690 to 1040 nm. The excitation wavelength was 850 nm. A green filter (495–540 nm) was used for GFP, and a red filter (575–645 nm) was used for RFP. A 25× objective (XLPLN25xWMP2) was used for this experiment. During the real-time imaging experiment, the mouse was placed on a staged heated to 37 °C and anesthetized with isoflurane anesthesia. The blood oxygen level and heart rate of the mouse were monitored during the experiment.

In order to compare results from this experiment with the traditional in vivo flow cytometry results, Fig. 1 was calculated by integrating the fluorescence signals from a fixed region in the blood vessels using the time-lapse images in which CTCs could be identified from the images, whereas the control data were calculated from the region at the edge of the same blood vessel. Each spike in Fig. 1 represents passing a single CTC. For visualization purposes, blood vessels were stained with FITC-dextran (shown in Fig. 2) and Evans blue (shown in Fig. 5). For the spontaneous CTC models in Fig. 3, 4, 6, and 7, no blood vessel staining dye was used. In order to observe the CTC number in Fig. 3, we imaged blood vessels at the edges of solid tumor from the same mouse in such way that the fluorescence signals from the tumors were just outside the field of view. In this experiment, we imaged the same group of three mice for 8 weeks. We tried our best to image the same area for each mouse at different weeks in which we imaged the blood vessels near the tumor boundary with similar blood vessel patterns and diameter while the size of tumor grew. No marker for imaging area was used in this experiment. In order to label a subpopulation of CTCs in vivo, antibody conjugated quantum dots (100 μl, 0.2 μM) were administered via tail vein injection using a single-use insulin syringe (1 ml, 30G, Omnican, B Braun) at a speed of roughly 10 μl/s. Image acquisition started 5 min after in vivo labeling.

### Statistical analysis and image reconstruction

All statistical analyses were conducted using Imaris 8.1 (Bitplane) and Origin 8.0. Three-dimensional images were reconstituted by Imaris 8.1.

## Additional file

**Additional file 1: Figure S1.** The two photon images of Evans blue in the blood vessels on the earlobe for (**a**) a 50-µm and (**b**) a 100-µm blood vessel. White signals are from the second harmonic generation indicating the boundary of the blood vessels. (**c**) Integrated fluorescence intensity of a section in the blood vessels. **Figure S2.** (**a**) Photos of mice with tumors on earlobes eight weeks after tumor cell inoculation. (**b**) IVIS image of tumors (BXPC3-RFP) grown on the earlobes of mice eight weeks after subcutaneous injection. (**c**) IVIS image of different organs. Metastatic sites can be found in the stomach and intestines. **Figure S3.** The average diameter of small capillaries in our tumor model was measured to be approximately 5 µm. **Figure S4.** Fibrillar collagen networks can be visualized second harmonic generation, which can be classified into two categories: elongated or curled. The average width of curled fibers was measured to be less than 2 µm, whereas that of the elongated fibers was approximately 4 µm. **Figure S5a.** The number of detected CTC/min in the blood vessels near the solid tumors on the earlobe at different time points post-inoculation. **Figure S5b.** The volume of solid tumors at different time points (n = 3). **Figure S6.** The velocity of CTCs and platelets, which were simultaneously imaged by labeling the platelets with anti-CD41-conjugated quantum dots.

**Additional file 2: Movie M1.** CD24+ cells (green) are moving in a blood vessel.

**Additional file 3: Movie M2a.** A CD24+ cell (green) is moving across the blood vessel wall.

**Additional file 4: Movie M2b.** Enlarged view of the CTC on the sidewall of blood vessel. The trajectory of the CD24+ is indicated.

**Additional file 5: Movie M3.** Movement of CD133+ CTC in the blood vessels. The red signals are from the anti-CD133 conjugated quantum, dots and the green signals are from the CTCs expressing green fluorescent proteins.

**Additional file 6: Movie M4.** Movement of palettes (red) and CTCs (green) in the blood vessels. For visualization, the trajectories of CTCs are highlighted by green traces in the movie.

**Additional file 7: Movie M5.** 3D microenvironment around the solid tumor. Green: blood vessels, red: cancer cells, white: ECM.
