# Supplementary material for: Real-time in vivo imaging of subpopulations of circulating tumor cells using antibody conjugated quantum dots
Source: J Nanobiotechnology. 2019 Feb 6;17:26. doi: 10.1186/s12951-019-0453-7 (PMC6364392; doi:10.1186/s12951-019-0453-7)
Supplement: Supplementary file 1 — Additional file 1: Figure S1. The two photon images of Evans blue in the blood vessels on the earlobe for (a) a 50-µm and (b) a 100-µm blood vessel. White signals are from the second harmonic generation indicating the boundary of the blood vessels. (c) Integrated fluorescence intensity of a section in the blood vessels. Figure S2. (a) Photos of mice with tumors on earlobes eight weeks after tumor cell inoculation. (b) IVIS image of tumors (BXPC3-RFP) grown on the earlobes of mice eight weeks after subcutaneous injection. (c) IVIS image of different organs. Metastatic sites can be found in the stomach and intestines. Figure S3. The average diameter of small capillaries in our tumor model was measured to be approximately 5 µm. Figure S4. Fibrillar collagen networks can be visualized second harmonic generation, which can be classified into two categories: elongated or curled. The average width of curled fibers was measured to be less than 2 µm, whereas that of the elongated fibers was approximately 4 µm. Figure S5a. The number of detected CTC/min in the blood vessels near the solid tumors on the earlobe at different time points post-inoculation. Figure S5b. The volume of solid tumors at different time points (n = 3). Figure S6. The velocity of CTCs and platelets, which were simultaneously imaged by labeling the platelets with anti-CD41-conjugated quantum dots. [file 12951_2019_453_MOESM1_ESM.pdf]

Supplementary Information

Real-Time *in vivo* Imaging of subpopulations of Circulating Tumor Cells Using Antibody Conjugated Quantum Dots

Chiung Wen Kuo, Di-Yen Chueh, Peilin Chen*

Research Center for Applied Sciences, Academia Sincia, Taiwan

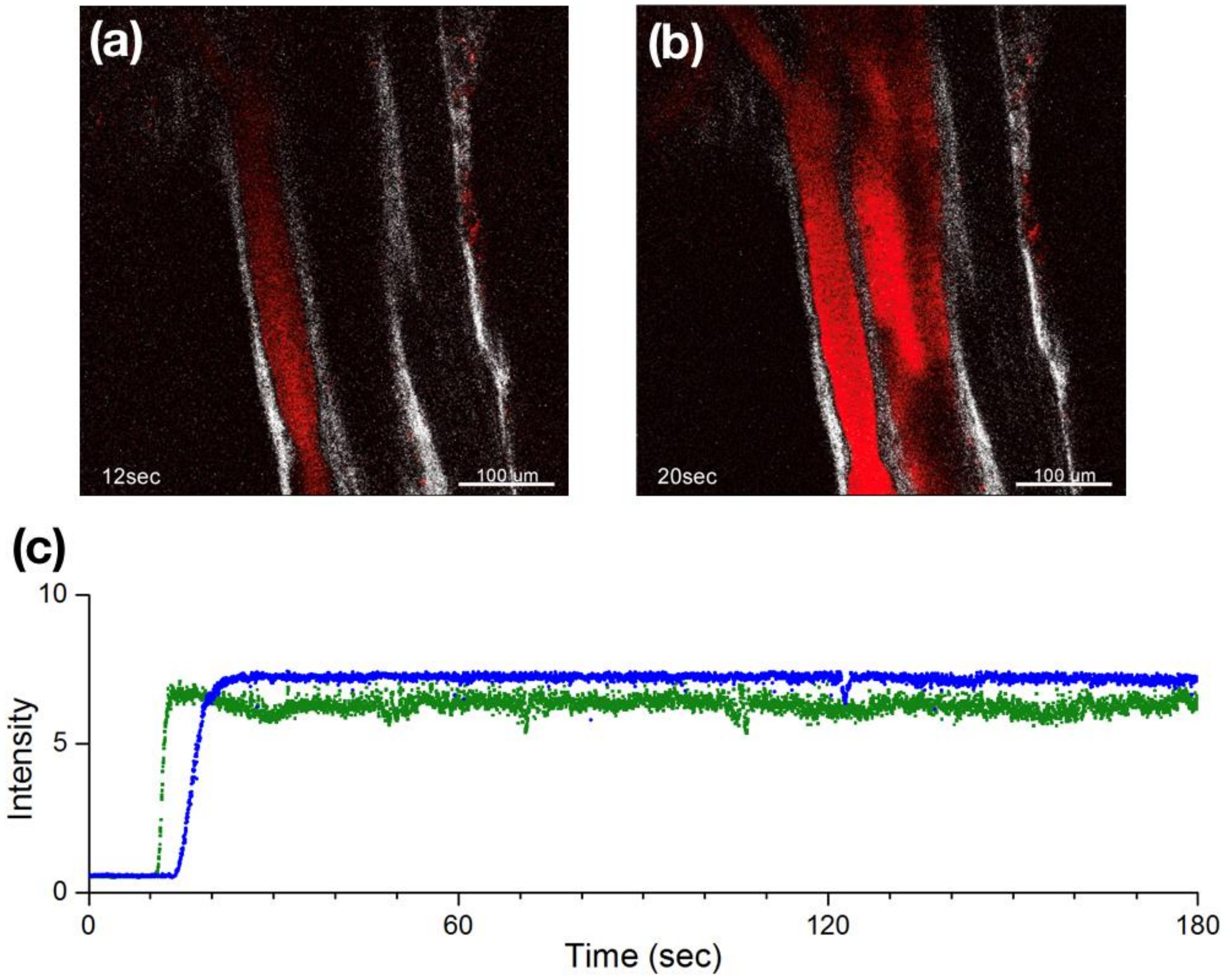

Figure S1. The two photon images of Evans blue in the blood vessels on the earlobe for (a) a 50-μm and (b) a 100 μm blood vessel. White signals are from the second harmonic generation indicating the boundary of the blood vessels. (c) Integrated fluorescence intensity of a section in the blood vessels.

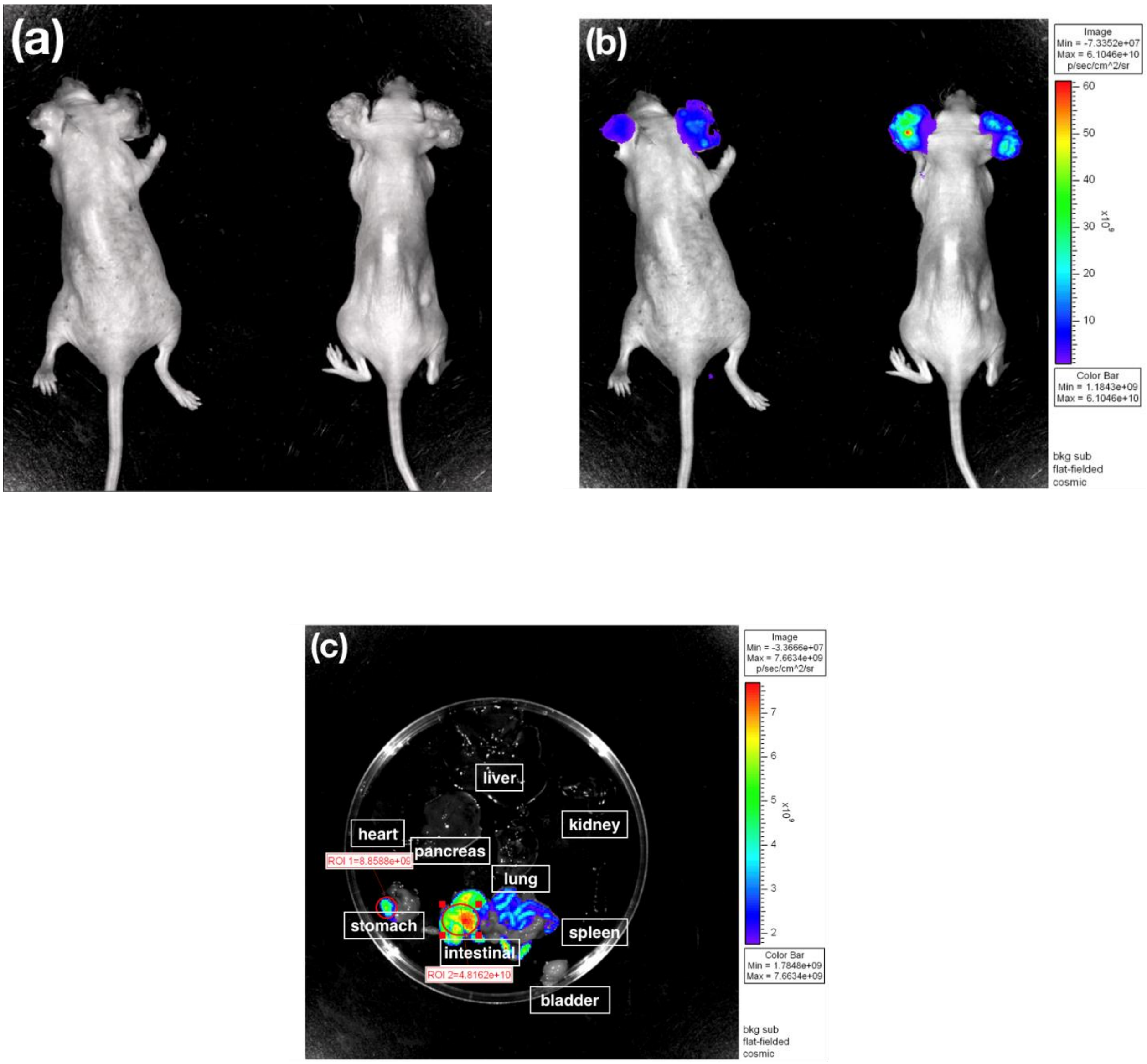

Figure S2. (a) Photos of mice with tumors on earlobes eight weeks after tumor cell inoculation. (b) IVIS image of tumors (BXPC3-RFP) grown on the earlobes of mice eight weeks after subcutaneous injection. (c) IVIS image of different organs. Metastatic sites can be found in the stomach and intestines.

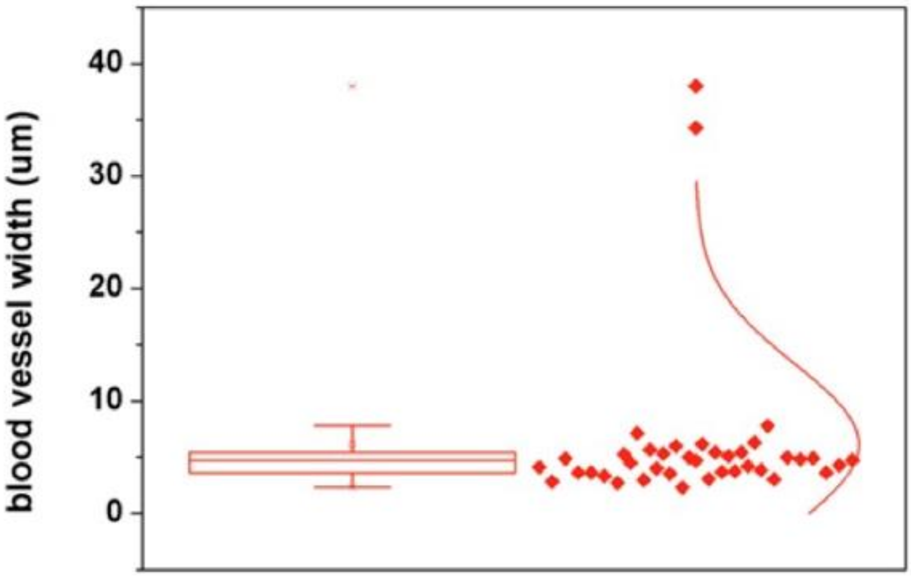

Figure S3. The average diameter of small capillaries in our tumor model was measured to be approximately 5 μm

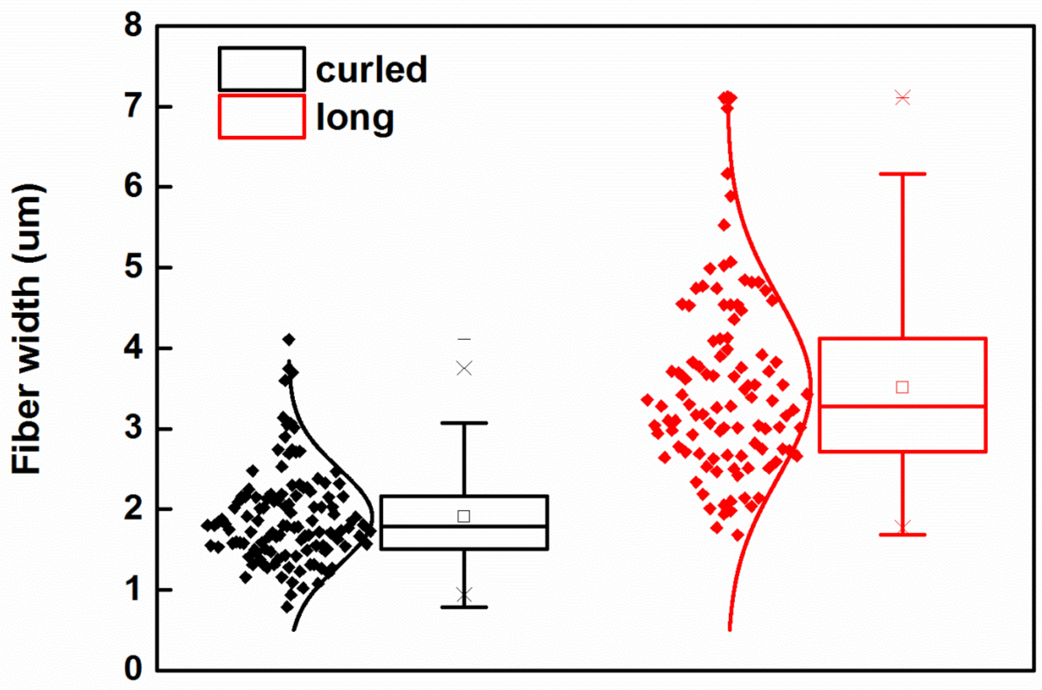

Figure S4. Fibrillar collagen networks can be visualized second harmonic generation, which can be classified into two categories: elongated or curled. The average width of curled fibers was measured to be less than 2 µm, whereas that of the elongated fibers was approximately 4 µm.

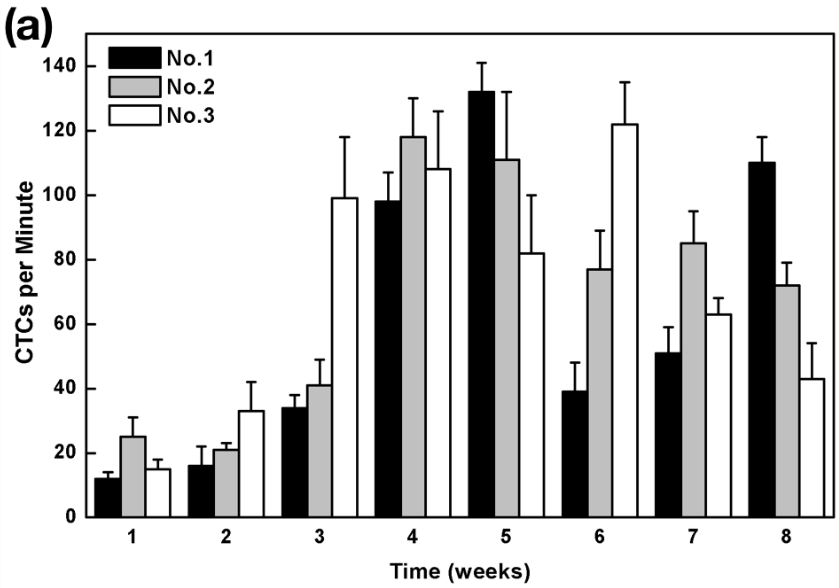

Figure S5a. The number of detected CTC/min in the blood vessels near the solid tumors on the earlobe at different time points post-inoculation.

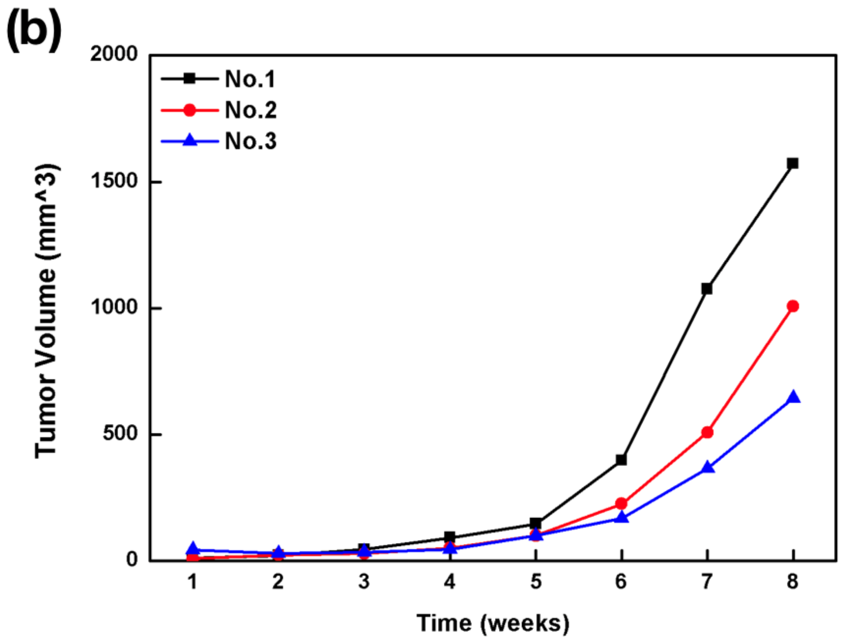

Figure S5b. The volume of solid tumors at different time points. (n = 3)

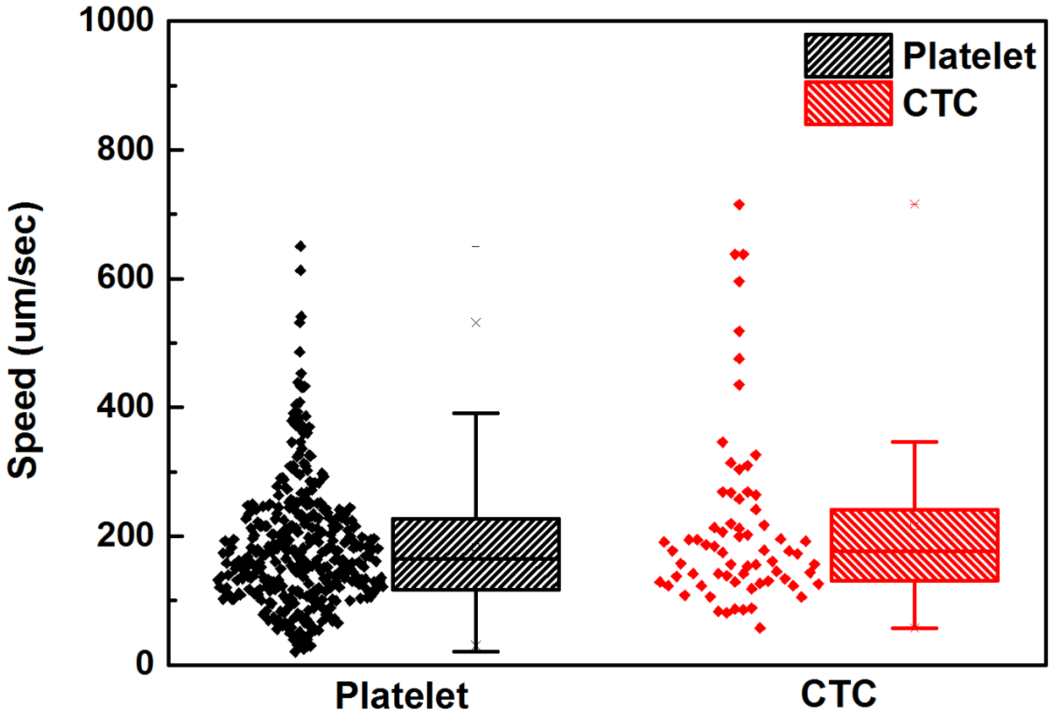

Figure S6. The velocity of CTCs and platelets, which were simultaneously imaged by labeling the platelets with anti-CD41-conjugated quantum dots.
