# Supplementary figures and images for: Real-time in vivo imaging of subpopulations of circulating tumor cells using antibody conjugated quantum dots
Source: J Nanobiotechnology. 2019 Feb 6;17:26. doi: 10.1186/s12951-019-0453-7 (PMC6364392; doi:10.1186/s12951-019-0453-7)

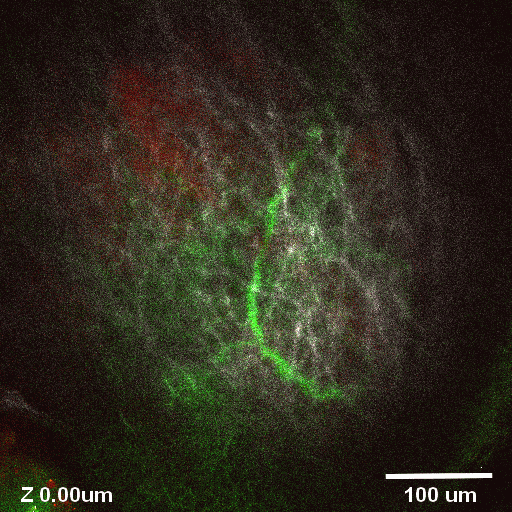

Supplement: Supplementary file 7 — Additional file 7: Movie M5. 3D microenvironment around the solid tumor. Green: blood vessels, red: cancer cells, white: ECM. [file 12951_2019_453_MOESM7_ESM.gif]
